# Exploring the costs, consequences and efficiency of three types of palliative care day services in the UK: a pragmatic before-and-after descriptive cohort study

**DOI:** 10.1186/s12904-020-00624-y

**Published:** 2020-08-07

**Authors:** Paul Mark Mitchell, Joanna Coast, Gareth Myring, Federico Ricciardi, Victoria Vickerstaff, Louise Jones, Shazia Zafar, Sarah Cudmore, Joanne Jordan, Laurie McKibben, Lisa Graham-Wisener, Anne M. Finucane, Alistair Hewison, Erna Haraldsdottir, Kevin Brazil, W. George Kernohan

**Affiliations:** 1 Health Economics Bristol, Population Health Sciences, University of Bristol, Bristol, UK; 2 Department of Statistical Science, University College London, London, UK; 3 Marie Curie Palliative Care Research Department, University College London, London, UK; 4 School of Nursing, Institute of Clinical Sciences, University of Birmingham, Birmingham, UK; 5 Division of Nursing, Queen Margaret University, Edinburgh, UK; 6 Centre for Clinical Brain Sciences, University of Edinburgh, Edinburgh, UK; 7 School of Health, Wellbeing and Social Care, The Open University, Milton Keynes, UK; 8 Institute of Nursing and Health Research, Ulster University, Newtownabbey, UK; 9 Marie Curie Hospice, Belfast and School of Psychology, Queen’s University Belfast, Belfast, UK; 10 Marie Curie Hospice, Edinburgh and Usher Institute, The University of Edinburgh, Edinburgh, UK; 11 St Columba’s Hospice, Edinburgh, UK; 12 School of Nursing and Midwifery, Queen’s University Belfast, Belfast, UK

**Keywords:** Palliative care day services, Costs, Health economics, Quality of life, End of life

## Abstract

**Background:**

Palliative Care Day Services (PCDS) offer supportive care to people with advanced, progressive illness who may be approaching the end of life. Despite the growth of PCDS in recent years, evidence of their costs and effects is scarce. It is important to establish the value of such services so that health and care decision-makers can make evidence-based resource allocation decisions. This study examines and estimates the costs and effects of PCDS with different service configurations in three centres across the UK in England, Scotland and Northern Ireland.

**Methods:**

People who had been referred to PCDS were recruited between June 2017 and September 2018. A pragmatic before-and-after descriptive cohort study design analysed data on costs and outcomes. Data on costs were collected on health and care use in the 4 weeks preceding PCDS attendance using adapted versions of the Client Service Receipt Inventory (CSRI). Outcomes, cost per attendee/day and volunteer contribution to PCDS were also estimated. Outcomes included quality of life (MQOL-E), health status (EQ-5D-5L) and capability wellbeing (ICECAP-SCM).

**Results:**

Thirty-eight attendees were recruited and provided data at baseline and 4 weeks (centre 1: *n* = 8; centre 2: *n* = 8, centre 3: *n* = 22). The cost per attendee/day ranged from £121–£190 (excluding volunteer contribution) to £172–£264 (including volunteer contribution) across the three sites. Volunteering constituted between 28 and 38% of the total cost of PCDS provision. There was no significant mean change at 4 week follow-up from baseline for health and care costs (centre 1: £570, centre 2: -£1127, centre 3: £65), or outcomes: MQOL-E (centre 1: − 0.48, centre 2: 0.01, centre 3: 0.24); EQ-5D-5L (centre 1: 0.05, centre 2: 0.03, centre 3: − 0.03) and ICECAP-SCM (centre 1:0.00, centre 2: − 0.01, centre 3: 0.03). Centre costs variation is almost double per attendee when attendance rates are held constant in scenario analysis.

**Conclusions:**

This study highlights the contribution made by volunteers to PCDS provision. There is insufficient evidence on whether outcomes improved, or costs were reduced, in the three different service configurations for PCDS. We suggest how future research may overcome some of the challenges we encountered, to better address questions of cost-effectiveness in PCDS.

## Background

Palliative care day services (PCDS) have developed to support people with life-limiting illness, who are able to attend a centre on a regular basis for symptom management, and emotional, psychological and social support [1, 2]. Typically, PCDS provide some core support services and respite for family carers [3]. PCDS in the UK are under the purview of four Departments of Health across the constituent countries: England, Scotland, Wales and Northern Ireland, but are not directly managed by them. They are delivered by organisations (typically charities) engaged to provide services. Although professional codes of practice apply across the UK and National Care Standards cover management, staff, and premises [4–6], as a devolved responsibility, health and social care service provision differs across the four countries, which contributes to local variation in how PCDS are delivered [7, 8].

Despite the growth of PCDS over the past 20 years in the UK, evidence of their costs and effects is limited. Where there is evidence, it has been collected in London and the South East of England in the main, and there is considerable variation in cost estimates for many types of palliative care services [9]. For example, a study conducted in 1999 involving five centres in Southern England found that PCDS cost around £79 (2018 value) per person per day, rising to £109 (2018 value) if unfunded resources (volunteer time) were included [10]. More recent estimates in a 2010/11 single-centre study in London put the cost of a day hospice attendance (excluding volunteer time) at £158 (2018 value) [11].^1^

It is unclear whether PCDS acts as a substitute for or a complement to other services to meet unmet need, an important issue when considering the impact of service on total costs. In one prospective quasi-experimental study of a hospice in the South East of England, PCDS were found to supplement rather than replace or reduce provision of other community and hospital services [12], and it did not provide details of any additional costs of day services used by attendees. Earlier work has, however, suggested that PCDS might substitute for home nursing or GP care and that PCDS provided in 1999 were not available elsewhere [10]. Quantitative data on the effectiveness of PCDS is also scarce [2].

We set out to examine and estimate the costs and effects of PCDS across a broader geographical area, to inform a more comprehensive view of PCDS across the UK.

## Methods

### Setting

We chose three services where dedicated research staff were available and had existing links to the research team. These links enabled an initial mapping exercise to be conducted to understand how these services were configured. This background information highlighted commonalities and differences between the services across the three sites and provided the context for the planning of the study design. We thus recruited attendees from three hospices delivering PCDS, one in each from England, Scotland and Northern Ireland. Each service offered a different mix of medical, nursing and allied healthcare, alongside social and psychological support. Centres operated between three to 5 days per week, with 12–15 places available per day and an average mean daily attendance of between 6 and 12 people [13].

### Study design

A pragmatic before-and-after descriptive cohort study was conducted involving people attending PCDS and those nominated by them as close persons, namely family and their close friends or informal carers [14]. To assure quality and safety, research governance approval was obtained at each hospice/centre (both terms used interchangeably here). Research ethics approval was granted by the NHS Health Research Authority West Midlands - Solihull Research Ethics Committee (reference: 17/WM/0100).

### Sample

Eligible participants were people consecutively referred to PCDS as out-patients or as part of a supportive approach to in-patient care. Inclusion was dependent on sufficient performance and cognitive functioning scores as assessed by nursing staff during attendees’ first visit to the PCDS centre using clinical judgement and validated tools (e.g. we excluded individuals with an Australian Karnosky Performance Scale Index ≤40 [15], or an ECOG Scale of Performance Status ≥3 [16], or an Abbreviated Mental Test ≤6 [17]). Participants were given the choice of completing the measures independently, together with a nominated close person, or with the help of a researcher. People were not eligible to participate if they had insufficient command of the English language to complete consent and data requirements, were aged under 18 or, to focus upon the intended population living at home, if they had been referred from a care home. Informed consent was given by attendees and close persons prior to data collection. A Participant Information Sheet and Letter of Introduction about the study were provided and discussed with a researcher before participants agreed to take part in the study and sign a consent form. Thereafter, a ‘process’ approach was adopted, whereby patient consent was confirmed on a continuing basis throughout the study [18]. Attendees were asked to nominate a close person and were given a letter, which provided an introduction to the study, to pass on to their close person. Participation in the study was not dependent on the recruitment of a close person.

### Data

To achieve the study objectives and based upon prior review of PCDS attendance, 9 months were allocated for data recruitment to achieve a sample size of 113 attendees and 113 close persons across the three centres at baseline (centre 1 *n* = 45, centre 2 *n* = 27 and centre 3 *n* = 41), with 50% of participants expected to remain in the study for 12 weeks (i.e. *n* = 57). This was not based on a specific sample size calculation due to the study design, but was based on what was believed to be pragmatically achievable across the three centres (i.e. 50% of all new attendees in each centre over 9 months), as well as drawing from recruitment undertaken in similar settings previously [19, 20].

PCDS attendees and close persons provided basic demographic information and data on health and care usage in the previous month using three adapted versions of the Client Service Receipt Inventory (CSRI) [21], to reflect the different services at the three centres and allow for variation in job role descriptions in England, Northern Ireland and Scotland. Attendance at PCDS ranged from 8 to 12 weeks duration. Attendees and close persons provided data at up to four time points (baseline, 4 weeks, 8 weeks and, where possible, at 12 weeks follow-up). Quality of life, health status and capability wellbeing measures were collected using the tools below:

- An expanded version of the McGill Quality of Life Questionnaire (MQoL-E), scored on a 0–10 worst-best quality of life scale, featuring eight subscales (physical, psychological existential, social, burden, environmental, cognition, healthcare) across 20 items. The MQOL-E was designed to comprehensively measure the subjective quality of life of a person with a life-threatening illness [22];
- Generic health status was measured using the EQ-5D-5L, containing five dimensions (mobility, self-care, usual activities, pain/discomfort and anxiety/depression) across five levels of severity problems [23]. Values for EQ-5D-5L are generated from the EQ-5D-3L UK general population crosswalk [24, 25]. EQ-5D-5L values are anchored on a 0–1 scale, representing dead - perfect health [26].
- A measure specifically developed to capture the benefits of end of life care in economic evaluation, the ICECAP-SCM, has seven domains covering choice/having a say in decision-making, love and affection/being with people who care, freedom from physical suffering, freedom from emotional suffering, dignity and self-respect, support and preparation [27]. The ICECAP-SCM main effects tariff from the general UK population was applied. ICECAP-SCM values are anchored on a 0–1 scale, representing no capability at the end of life – full capability at the end of life [28].

Two measures, used primarily in health economic analysis, were included, because the EQ-5D-5L has been shown to have little in common with outcomes relevant for palliative care [29] and patients and close persons feel outcomes such as the opportunity for a good death are more appropriate (e.g. ICECAP-SCM) [30].

There are two broad types of costing approaches: top-down or bottom-up. A top-down approach involves identifying the total costs of a programme or service and apportioning those costs to the components involved in its delivery. A bottom-up approach identifies and measures individual components of a service in a disaggregated way [31]. Both approaches were used, however, given the smaller than anticipated sample size, it was not possible to determine the costs of the PCDS from the prospective data alone. Consequently, a scoping exercise of service provision and use at the three PCDS sites was undertaken in 2015 [13]. The findings were combined with the cohort data and information provided by the key contacts at each centre to estimate the total cost per attendee/day at each PCDS using the top-down approach. Health and care costs reported in the CSRI were taken from the cohort data only using bottom up costing.

The economic valuation of resource use data required a combination of methods. Costs for the PCDS were based on cost per attendance. This was estimated using information on resource use available in the hospices, including information on the delivery of day hospice, such as staffing and volunteer mix. Health care and social services resource use, staffing, volunteering and mileage was valued using the Unit Costs of Health and Social Care 2018 edition [32]. Minimum wage costs were applied when volunteering roles were unavailable as an equivalent market rate for such roles, a common approach when calculating the economic cost of volunteer time [33]. All costs are valued in pound sterling (GBP) in the year 2018.

### Data analysis

Due to a smaller than anticipated sample, the analysis is concentrated on examination of complete cases of PCDS attendees at the three centres. The differences in costs and outcomes at four-week follow up, compared to baseline and comparison of costs per day attendance at PCDS across the three centres, are analysed. This was done to focus on the most complete data available. Paired t tests [34] were conducted to assess the difference in mean outcome and cost data at the four-week follow up compared to baseline at the three centres. Significance level was set to 5 and 95% confidence intervals were estimated for the differences between paired costs and outcomes at the centre level. A scenario analysis, examining cost per attendee/day using three hypothetical attendance rates (100, 80 and 60%) was conducted to investigate the variation in costing within and across the three PCDS.

## Results

Table 1 describes the staffing and volunteer composition of the three PCDS centres. It also includes information on the operation of PCDS, in terms of days open, places available for attendees per day, and the attendance rates of the three centres in 2015. It details the staff and volunteer composition of the three centres in 2018/19.

**Table 1:** Composition of three Palliative Care Day Services (PCDS) centres in the UK

	Centre 1	Centre 2	Centre 3	Notes
**PCDS Operation in 2015**				A
Days in operation per week	5	3	5	A
Sites where PCDS in operation	3	1	1	A,B
Places available per day	15	12	15	A
Attendance rate	75%	56%	60%	A
**Staff in 2018/19 (Proportion of FTE)**				C
Manager - social worker			1.0	D
Manager - nurse	1.0			D
Manager - allied health professional		0.6		D
Associate Specialist Doctor	0.4			D
Nurse consultant	0.2			D
Social worker	0.6			D
Palliative care nurse			1.0	D
Occupational therapist	1.0	0.6	0.2	D
Physiotherapist	1.0		0.2	D
Registered nurse	1.0	0.6	1.0	D
Rehabilitation assistant		0.6		D
Health Care Assistant	1.0			D
Secretary	1.0			D
**Volunteers in 2018/19 (Proportion of FTE)**				
Complementary therapists	2.0	0.2	0.4	D
Drivers	3.0	3.6	2.4	E
Hospitality	3.0	1.8	2.8	E
Hairdresser		0.25		E

A. Operational data from a mapping exercise of the three centres in 2015 - see [13]; B. Centre 1 operates 3 days a week at the main site and at two satellite units running one day a week each; Centre 2 now operates 5 days a week, with a satellite unit running two days a week; C. Staff and volunteer numbers providing from the three centres in 2018/19; D. From [32]; *E. minimum* wage rate for 2017/18 applied (£15,269 per year): source Office for National Statistics; *FTE* full-time equivalent

Participants were recruited between June 2017 and September 2018 across the three centres (centre 1 from June to October 2017 and from July to September 2018; centre 2 from January to March 2018; centre 3 from January to June 2018). There were inconsistencies between start and finish time at each site, due to local differences in approval dates. Data collection at centre 1 was interrupted due to staff changes. Due to larger than anticipated recruitment difficulties, a decision was made during recruitment to stop data collection at 12 weeks for attendees and all data collection for close persons.

In total 56 attendees completed baseline data (centre 1 *n* = 16, centre 2 *n* = 9, centre 3 *n* = 31), 38 at 4 weeks (centre 1 *n* = 8, centre 2 *n* = 8, centre 3 *n* = 22), 31 at 8 weeks (centre 1 *n* = 6, centre 2 *n* = 7, centre 3 *n* = 18), and 16 at 12 weeks (centre 1 *n* = 1, centre 2 *n* = 6, centre 3 *n* = 9). Most attendees requested help from the researcher to complete the questionnaires. For close persons, the total sample at baseline was 22 (centre 1 = 9, centre 2 = 5, centre 3 = 8), 14 at 4 weeks (centre 1 = 4, centre 2 = 5, centre 3 = 5), 10 at 8 weeks (centre 1 = 3, centre 2 = 4, centre 3 = 3) and 9 at 12 weeks (centre 1 = 1, centre 2 = 5, centre 3 = 3). The results focus on attendee cases with complete data at baseline and 4 weeks (*n* = 38).

Table 2 reports descriptive statistics for the 38 participants for whom there was follow-up data at 4 weeks. Most participants were aged over 65 at the time of recruitment (age range in years for centre 1: 57–81; centre 2: 51–91; centre 3: 41–88), white, living in owner occupied homes, were married, did not live alone and had a cancer diagnosis. This is consistent with the wider population of those referred to hospice services [35]. The source of referral and the average time from diagnosis to commencing PCDS varied between the centres.

**Table 2:** Characteristics of attendees from the three PCDS centres included in analysis

	Centre 1	Centre 2	Centre 3	Total (%)
Sample size (n)	8	8	22	38 (100%)
Average mean age (years)	66	75	68	69
Male (%)	7	4	9	20 (53%)
White ethnicity (%)	6	8	19	33 (87%)
Married (%)	5	4	11	20 (53%)
Living alone (%)	3	4	6	13 (34%)
Owner occupied homes (%)	5	6	16	27 (71%)
Cancer diagnosis (%)	7	3	14	24 (63%)
Average time since diagnosis (years)	1	5	4	4
*Referral source (%)*				
Hospital consultant	8			8 (21%)
Hospice community nurse		4	4	8 (21%)
Clinical nurse specialist		3	5	8 (21%)
GP		1	1	2 (5%)
Respiratory service			5	5 (13%)
Specialist palliative care team			2	2 (5%)
Occupational therapist			2	2 (5%)
Within hospice referral			2	2 (5%)
Not available			1	1 (3%)

The mean cost per attendee/day ranged from £121 to £190 across the three centres (Table 3). Including volunteer costs raises the average cost per attendee/day to £172 to £264 across the three centres. Volunteering constituted between 28 and 38% of the total costs of PCDS provision.

**Table 3:** Cost per attendee/day at three Palliative Care Day Services in the UK

Centre	Costs excluding volunteer contribution	Costs including volunteer contribution
1	£190	£264
2	£164	£263
3	£121	£172

Table 4 reports changes in non-hospice health and care resource use and changes in outcome over 4 weeks. There were no statistically significant differences in the costs and outcomes between the three PCDS at follow-up. The direction of the mean changes in costs and outcomes across the centres, with the varied geographical locations of and differences in service provision, do not form a consistent pattern.

**Table 4:** Change in health and care resource use and attendee outcome at 4 week follow-up

Cost/outcome	Centre	n	Baseline	4 week	Change (95% CIs)
Health and care costs	1	7	£1508	£2078	£570 (−£1155, £2255)
	2	8	£1920	£793	-£1127 (−£2642, £388)
	3	22	£1187	£1252	£65 (−£946, £1076)
MQOL-E	1	8	7.11	6.64	−0.48 (−1.72, 0.76)
	2	8	6.88	6.89	0.01 (−0.70, 0.72)
	3	22	5.97	6.21	0.24 (−0.29, 0.77)
EQ-5D-5L	1	7	0.38	0.43	0.05 (− 0.19, 0.29)
	2	8	0.43	0.46	0.03 (−0.14, 0.19)
	3	22	0.59	0.56	−0.03 (− 0.17, 0.11)
ICECAP-SCM	1	8	0.87	0.87	0.00 (−0.07, 0.07)
	2	8	0.90	0.89	−0.01 (− 0.03, 0.02)
	3	22	0.80	0.83	0.03 (−0.02, 0.07)

*CIs* confidence intervals; MQOL-E is scored on a 0–10 worst-best quality of life scale; EQ-5D-5L is scored on a 0–1 dead-perfect health scale; ICECAP-SCM is scored on a 0–1 no capability at the end of life-full capability at the end of life scale

A scenario analysis of the cost per attendee/day estimated by varying attendance rates demonstrates a large variation in costs across scenarios and centres (Table 5). The costs for centre 1 are almost twice those of centre 3, whether volunteer contribution is included or not.

**Table 5:** Scenario Analysis of cost per attendee/day

	Excluding Volunteer Costs			Including Volunteer Costs		
Attendance Rate	Centre 1	Centre 2	Centre 3	Centre 1	Centre 2	Centre 3
100%	£142	£91	£73	£199	£149	£107
80%	£177	£114	£91	£247	£185	£131
60%	£236	£152	£121	£327	£244	£172

## Discussion

The mean cost of providing PCDS across the three centres ranged from £121 to £190 per attendee/day. The cost of providing PCDS is considerably higher when the value of volunteering is accounted for, raising it to between £172 to £264 per attendee/day (Table 3). There was also variation in how individual PCDS centres operationalised services, in particular, the staffing mix being considerably higher at centre 1 compared to centres 2 and 3, resulting in the cost per attendee/day at centre 1 being almost double those at centres 2 and 3, when attendance rates are held constant (Table 5). As illustrated in our scenario analysis (Table 5), one way of minimising the current cost per attendee/day and maximising the number of attendees accessing PCDS would be to increase attendance rates across all centres within existing staffing and volunteering resources. It would be important to ensure, however, that this did not reduce the quality of PCDS provided. The analysis from this cohort study is exploratory and we were unable to conclude whether PCDS reduces other health and care costs or improves outcomes, when using a generic health status measure (EQ-5D-5L), a capability wellbeing near the end of life measure (ICECAP-SCM) or a quality of life during a life-threatening illness measure (MQOL-E) (Table 4).

A strength of this study is the use of data from three centres with different service configurations serving local communities which together were reflective of the wider diverse UK population. All sites were research ready environments, with site-specific research governance procedures in place and had participated in previous research projects. The study is limited by the small sample size and it was not possible to compare the three services. The recruitment figures were affected by unforeseen delays in recruiting research staff, a delay in securing ethical approval, and well documented difficulties involved in recruiting to studies of palliative care [36].

The five main challenges we experienced to varying degrees were [37]:

1. ensuring participant identification through understanding the inclusion and exclusion criteria;
2. excessive participant burden in data collection;
3. limited staff time;
4. clinical deterioration in participants and
5. complexity in caregiver involvement.

(1) PCDS operates in a flexible way with more repeat visits than new cases: here, we limited our inclusion of new attendees at their first attendance, aiming to capture each episode of care from the start, leading to a slow start as we did not include current caseload but waited to recruit new referrals. (2) It is inevitable that data capture requires time and effort by participants and this affected the data collection. Indeed, we set out to obtain several repeated measures and this may have been too difficult as participants’ health deteriorated and it seemed attrition was inevitable. However (3) shortage of staff time and (4) client deterioration were not deemed to cause concern with PCDS, a relatively well-staffed and early-stage intervention. (5) Close-person involvement was difficult to achieve because of caregiver distress, the practicalities of obtaining dual consent, and a lack of recognition on the part of the close persons of the value of the study. In addition, many could not find time to participate.

The accuracy of the costing was also affected by the small sample size, in particular in centres 1 and 2, so data were drawn from 2015 PCDS models of service delivery [13] to compensate for the limited data collected. Careful interpretation of results is essential given potential biases arising from missing data [38]. There are several options for managing missing data in economic analyses [39] but the sample size in this study precluded their application. Analysis of cohort study data was conducted using complete case analysis.

A systematic review on the costs of palliative care in the UK highlighted the variation in estimates in different studies [9]. Our study found that costs for PCDS also vary across centres and across the UK, with differences in staff mix, volunteer mix and attendance rates all identified as elements of this variation. Our study suggests the costs of providing PCDS may have been underestimated in earlier work [10, 11]. However, it does confirm the extensive and significant role volunteers play in the provision of PCDS [10], even though volunteer time is not routinely included in health and care economic evaluations in the UK [40]. Volunteers’ roles in PCDS are wide ranging, and include complementary therapy, beauty therapy/hairdressing and pastoral/faith based care services [41], offering a distinct contribution, and in many cases assuming the characteristics of a paid employee [42]. Exclusion of volunteers from costing PCDS underestimates both the economic costs of providing PCDS and the value of the volunteers’ time.

The gap between the number of staff needed in health and care in England alone and those available could reach more than 350,000 by the year 2030 if the number of staff leaving the workforce early continues and recruitment of newly trained staff and international recruits does not increase [43]. This also has implications for hospice services and has been identified as a key priority by Hospice UK which is committed to working with hospices and partner organisations to develop sustainable solutions to meet these workforce challenges [44]. This suggests the contribution of volunteers to hospice services may become an even more important issue in workforce planning. When we included the value of volunteering in the costs for providing PCDS, we found that 28–38% of costs per attendee/day was volunteer contribution to PCDS delivery at these three centres across the UK. It is important to discover if this constitutes a ‘critical mass’ in terms of the staff skill mix in such services, or if there is scope for expansion. Indeed, a recent study concluded there is scope for hospices to develop strategic aspects of volunteering through greater community engagement and involvement [45]. Moreover, it was recognised some years ago that there was a need to increase recruitment to expand the volunteer workforce, and that hospices need to be supported by more effective and extensive deployment of volunteers [46]. However, it is interesting to note that in a later workforce policy statement [44] there was no indication of how this will be achieved. Our findings provide useful detail for those addressing workforce issues at a policy and practice level.

No significant change in costs and effects at any of the three centres after 4 weeks were indicated by the cost and outcomes data (Table 4), but these results must be regarded as exploratory only. Thus, one key aspect yet to be determined is whether the provision of PCDS represents a good investment for improving attendee outcomes for PCDS providers or by reducing costs for health and care services as a whole. This question is not only important to commissioners and providers of PCDS in offering value for money and to attendees who may benefit from such services, but also to ensure that the contribution of PCDS staff and volunteers is being managed effectively to maximise the benefit of the service they offer. Further work is needed to establish the benefits and costs of PCDS. For example, quasi-experimental designs have been used in a similar setting where randomised controlled trials were not feasible, and similar designs could potentially be used to evaluate PCDS [12]. Future research would also be beneficial to assess the costs and outcomes for close persons of PCDS attendees to see if benefits of PCDS extend beyond the attendee [47].

## Conclusions

This study highlights the important contribution of volunteers to PCDS provision. We found there was insufficient evidence to draw conclusions about whether outcomes improve, or costs reduce, in the three different service configurations for PCDS. We provide suggestions for future research that is needed to overcome some of the challenges we encountered in this study to better address questions concerning the cost-effectiveness of PCDS.

## Data Availability

The data that support the findings of this study are available upon reasonable request from the corresponding author [PM]. The data are not publicly available due to them containing information that could compromise research participant privacy.

## Abbreviations

CSRI: Client Service Receipt Inventory
MQOL-E: McGill Quality of Life Questionnaire - Expanded
PCDS: Palliative care day services

## Footnotes

1 Costs taken from previous studies are adjusted to 2018 net present values using the UK Treasury Gross Domestic Product (GDP) deflator.
